# Long‐term disease course of two patients with multiple sulfatase deficiency differs from metachromatic leukodystrophy in a broad cohort

**DOI:** 10.1002/jmd2.12189

**Published:** 2020-12-08

**Authors:** Stefanie Beck‐Wödl, Christiane Kehrer, Klaus Harzer, Tobias B. Haack, Friederike Bürger, Dorothea Haas, Angelika Rieß, Samuel Groeschel, Ingeborg Krägeloh‐Mann, Judith Böhringer

**Affiliations:** ^1^ Institute of Medical Genetics and Applied Genomics University of Tübingen Tübingen Germany; ^2^ Department of Neuropediatrics University Children's Hospital Tübingen Germany; ^3^ Metabolic Centre University Children's Hospital Heidelberg Germany

**Keywords:** multiple sulfatase deficiency, *SUMF1*, metachromatic leukodystrophy, lysosomal storage disorder, natural course, arylsulfatase A deficiency

## Abstract

Multiple sulfatase deficiency (MSD) is a lysosomal storage disease caused by a deficiency of formylglycine‐generating enzyme due to *SUMF1* defects. MSD may be misdiagnosed as metachromatic leukodystrophy (MLD), as neurological and neuroimaging findings are similar, and arylsulfatase A (ARSA) deficiency and enhanced urinary sulfatide excretion may also occur. While ARSA deficiency seems a cause for neurological symptoms and later neurodegenerative disease course, deficiency of other sulfatases results in clinical features such as dysmorphism, dysostosis, or ichthyosis. We report on a girl and a boy of the same origin presenting with severe ARSA deficiency and neurological and neuroimaging features compatible with MLD. However, exome sequencing revealed not yet described homozygosity of the missense variant c.529G > C, p.Ala177Pro in *SUMF1*. We asked whether dynamics of disease course differs between MSD and MLD. Comparison to a cohort of 59 MLD patients revealed different disease course concerning onset and disease progression in both MSD patients. The MSD patients showed first gross motor symptoms earlier than most patients with juvenile MLD (<10th percentile of Gross‐Motor‐Function in MLD [GMFC‐MLD] 1). However, subsequent motor decline was more protracted (75th and 90th percentile of GMFC‐MLD 2 (loss of independent walking) and 75th percentile of GMFC‐MLD 5 (loss of any locomotion)). Language decline started clearly after 50th percentile of juvenile MLD and progressed rapidly. Thus, dynamics of disease course may be a further clue for the characterization of MSD. These data may contribute to knowledge of natural course of ultra‐rare MSD and be relevant for counseling and therapy.

**Synopsis**

This article provides information about differences in dynamics of disease course in arylsulfatase A deficient patients, comparing two individuals with MSD to a broad cohort of MLD patients.

## 1 INTRODUCTION

Ultra‐rare multiple sulfatase deficiency (MSD; OMIM #272200) is caused by pathogenic variants in *SUMF1*.^1^, ^2^, ^3^, ^4^, ^5^ *SUMF1* encodes formylglycine‐generating enzyme (FGE) (EC 1.8.3.7), which is essential for sulfatase activation.^6^ FGE deficiency influences the clinical MSD picture by effects of the respective affected sulfatases. Correlation with their remaining activity is missing, and even normal activity of single sulfatases occurs.^1^, ^2^, ^7^, ^8^, ^9^ Deficiency of arylsulfatase A (ARSA) (EC 3.1.6.8) (also due to pathogenic variants in *ARSA* or *PSAP* revealing metachromatic leukodystrophy [MLD; OMIM #250100] or SAP‐B deficiency [SAP‐B; OMIM #249900]) leads to accumulation of sulfatides causing neurodegeneration.^10^ Severe ARSA deficiency is a relatively constant finding in MSD causing MLD‐typical features with neurological symptoms (like spasticity, tremor, ataxia, or dysphagia) and loss of skills, both phenomena characterizing mainly the later disease stage.^11^ Deficiency of other sulfatases can result into dysmorphism, organomegaly, or dysostosis multiplex, resembling different mucopolysaccharidoses, like mucopolysaccharidosis II (M. Hunter; OMIM #309900), IIID (M. Sanfilippo‐D; OMIM #252940), or VI (Maroteaux‐Lamy‐Syndrom; OMIM #253200) for example due to deficiency of iduronate‐2‐sulfatase (EC 3.1.6.13), N‐acetylglucosamine‐6‐sulfatase (EC 3.1.6.14), or arylsulfatase B (ARSB) (EC 3.1.6.12), respectively.^8^ X‐linked ichthyosis (OMIM #308100) and chondrodysplasia punctata‐1 (OMIM #302950) are associated with deficiency of steroid sulfatase (EC 3.1.6.2)^12^ and arylsulfatase E (EC 3.1.6.1), respectively. Clinical MSD spectrum ranges from severe neonatal presentation to severe/mild‐infantile and milder juvenile types.^1^, ^2^, ^5^, ^11^, ^13^ Genotype‐phenotype‐correlation in MSD is discussed controversially.^1^, ^2^, ^3^, ^7^, ^13^, ^14^, ^15^ MSD spectrum of the age of onset, clinical signs, and genotypes is described in recent systematic literature reviews and natural history study.^1^, ^2^, ^7^ In this study we investigate how dynamics of the disease course differs between MLD and MSD—in addition to differences of clinical features and the biochemical profile. Therefore, we compared the long‐term disease dynamics of two children suffering from MSD to a broad cohort of MLD patients. For this approach, we referred to reliable tools describing neurodegenerative diseases, as scores for language^16^ and gross motor function.^17^, ^18^ The Gross‐Motor‐Function‐Classification in MLD (GMFC‐MLD) represents all clinically relevant disease stages from normal (level 0) to complete loss of gross motor function (level 6).^17^, ^18^ It can be used retrospectively, indispensably for data acquisition in rare diseases. It has been used for natural history studies,^7^, ^16^, ^18^, ^19^, ^20^, ^21^ and studies evaluating therapy.^22^, ^23^, ^24^, ^25^, ^26^, ^27^

## 2 MATERIAL AND METHODS

### 2.1 Clinical investigation

Neurological examination of patient 1 was done at the ages of 7.9 years, 9.3, 10.8, 12.8, 14.3, and 15.5 years, and of patient 2 at ages 8.8 and 16.0 years. Investigation included (partly repeated) electroneurography, neuroimaging, abdominal ultrasound, and X‐ray of the spine. Visual‐ or hearing tests and electrocardiography were not performed. For the assessment of language‐, swallowing‐ and gross motor function, standardized tools were used (expressive‐language‐function‐classification [ELFC‐MLD],^16^ eating‐and‐drinking‐ability‐classification‐system [EDACS],^28^ GMFC‐MLD,^17^, ^18^ gross‐motor‐function‐measure [GMFM‐88]^29^) (Supplementary Material S1). Neurologic and somatic exam features and medical complications of MSD according to Adang et al^7^ are shown in Supplementary Material S2. Long‐term disease course was followed up until the present (15 years, patient 1) or death (20 years, patient 2) by regular (non‐standardized) telephone contacts.

### 2.2 Genetic analysis

In both patients, molecular genetic analysis of all exons of *ARSA* and *PSAP* was performed by conventional Sanger sequencing according to standard protocols. Subsequently, exome sequencing was performed on genomic DNA from peripheral blood leukocytes of patient 1. Coding genomic regions were enriched with a SureSelect Human All Exon Kit V6 (Agilent technologies, Santa Clara, California) for subsequent sequencing as 2 × 125 bp paired end reads on an HiSeq2500 system (Illumina, San Diego, California). Generated sequences were analyzed using the megSAP pipeline (Supplementary weblinks). Clinical variant prioritization included different filtering steps (eg, MAF <0.1% in 1000 g, ExAC, gnomAD and in‐house database). Confirmation of the identified *SUMF1* variant in patient 1, diagnostic testing in patient 2, and carrier testing was done by Sanger sequencing. Genotype data and some clinical aspects of the two patients are published in a larger MSD cohort.^7^

### 2.3 Biochemical analysis

Sulfatase activities were measured in leukocytes and plasma according to standard methods.^30^, ^31^, ^32^, ^33^ Analysis of urinary sulfatides and glycosaminoglycans was performed according standard protocols^34^, ^35^, ^36^ (see Supplementary Material S3).

### 2.4 MLD cohort

Disease course of two MSD patients was compared with a cohort of 59 MLD patients (21 late‐infantile, 38 juvenile) who were recruited within the scope of the nationwide German research network LEUKONET. Disease onset before the age of 30 months was defined as “late‐infantile”, and between 30 months and 15 years as “juvenile”.^18^ Dynamics of motor and language decline in the MLD patients was previously published.^16^, ^18^, ^19^

## 3 RESULTS

### 3.1 Patient histories

**FIGURE 1:**
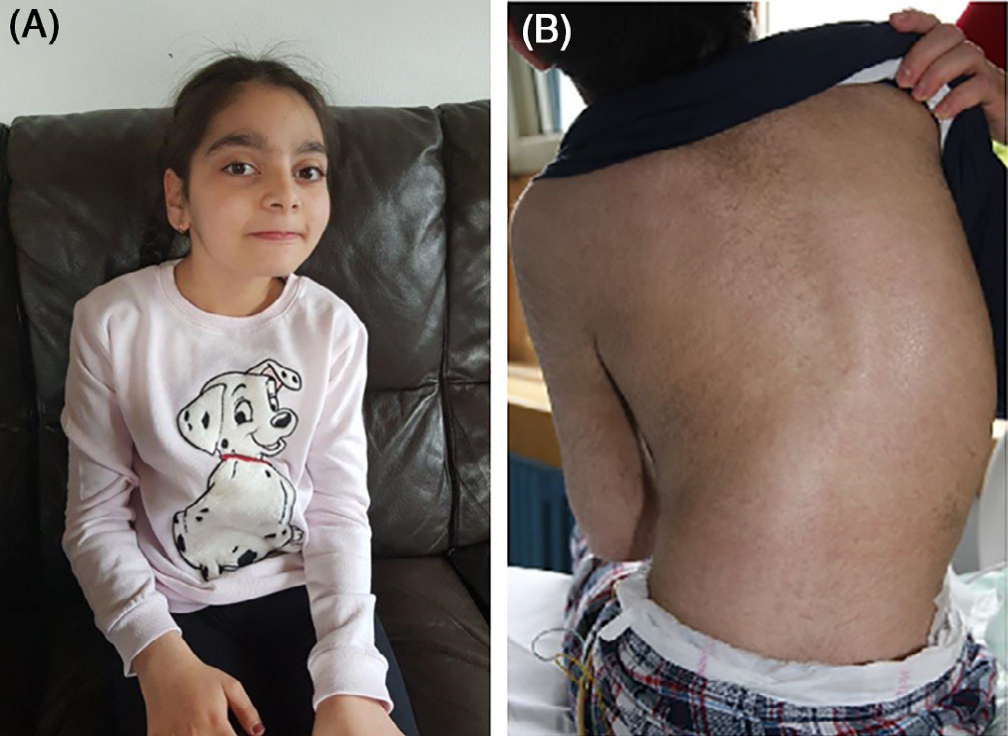
Photographs of two patients suffering from multiple sulfatase deficiency (MSD) due to homozygous missense change in *SUMF1* (c.529G > C). A, Patient 1 at the age of 14 years. B, Patient 2 at the age of 16 years; note scoliosis of the spine; the partly patchy skin is mainly due to ichthyosis

Both patients (Figure 1A,B) were firstborns of healthy first‐degree consanguineous parents originating from the same district of a village in Denizli province, Turkey. Both families meanwhile live in different towns in Germany. For detailed description of the patients and their disease course (birth data, infantile development, disease onset, clinical and neurological symptoms, and subsequent motor‐, cognition‐, and language decline) see Supplementary Material S1.

### 3.2 Clinical investigations

**FIGURE 2:**
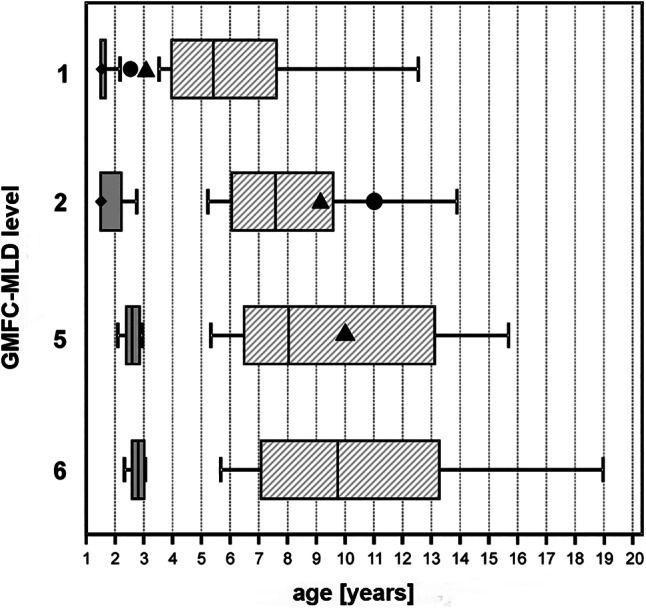
Dynamics of motor decline of patient 1 and patient 2 both suffering from multiple sulfatase deficiency (MSD) due to homozygous missense change in *SUMF1* in comparison to a cohort of 59 untreated patients (21 late‐infantile, 38 juvenile) with metachromatic leukodystrophy (MLD). Age at entry into a respective level of the Gross Motor Function Classification in Metachromatic Leukodystrophy (GMFC‐MLD) for late‐infantile (dark gray) and juvenile (hatched) forms combined.^18^ Whiskers indicate 10% and 90% percentiles, vertical indicates median. GMFC‐MLD level 1: First motor symptoms. GMFC‐MLD level 2: Loss of free walking. GMFC‐MLD level 5: Loss of gross motor function but head control preserved. GMFC‐MLD‐level 6: Loss of any locomotion as well as loss of any head‐ and trunk control. Patient 1 (circle) enters GMFC‐MLD level 1 at the age of 2,5 years, and patient 2 (triangle) at the age of 3 years. Patient 1 enters GMFC‐MLD level 2 at the age of 11 years and patient 2 at the age of 9 years. Patient 1 did not enter GMFC‐MLD level 5, and patient 2 enters GMFC‐MLF level 5 at the age of 10 years

Patient 1 and 2 showed first motor symptoms (GMFC‐MLD level 1) at the age between 30 and 36 months. Patient 1 lost independent walking (GMFC‐MLD level 2) at the age of 11 years, patient 2 at 9 years of age. Patient 2 showed loss of gross motor function with only head control preserved (GMFC‐MLD level 5) at the age of 10 years, while this did not occur in patient 1 in the observed period (aged 15 years). Time difference from GMFC‐MLD level 1 to level 2 was 8 years in patient 1 and 6 years in patient 2. Time difference from GMFC‐MLD level 2 to level 5 was 1 year in patient 2 and not reached in patient 1. Patients' gross motor abilities assessed by GMFC‐MLD levels in comparison to the MLD cohort are shown in Figure 2. First language decline (ELFC‐MLD 1) occurred 36 months after motor onset in patient 1 (aged 6 years) and 42 months in patient 2 (6.5 years). Complete loss of expressive language (ELFC‐MLD 4) occurred 8 and accordingly 1 year later (patient 1 aged 14 years, patient 2 aged 7.5 years). Regression and loss of language assessed by the ELFC‐MLD, and regression in eating and drinking assessed by the EDACS are listed in Supplementary Material S1. In both patients electroneurography revealed reduced motor and sensory nerve conduction velocities compatible with mixed axonal and demyelinating polyneuropathy (Table S1). Cerebral MRI showed a severe leukodystrophy pattern characteristic for MLD in both patients (MR‐MLD severity score 21 and 29 respectively, Figure S1). Ultrasonography revealed no organomegaly and X‐ray no bone alterations suggestive of mucopolysaccharidosis in both patients. None of the patients suffered from visual/hearing‐problems hampering everyday life. Both had short stature (third percentile), but no microcephalia.

### 3.3 Genetic findings

**FIGURE 3:**
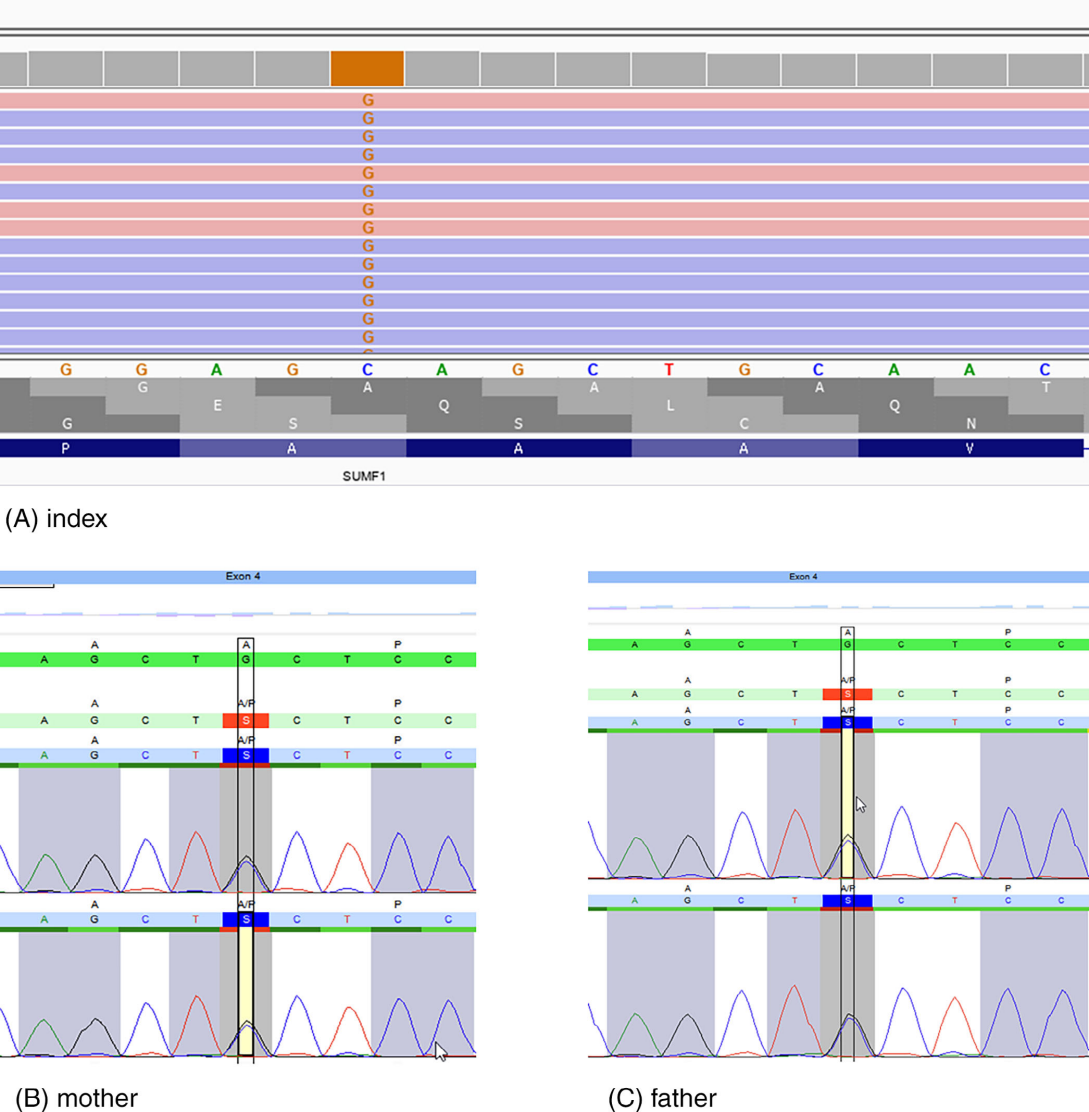
*SUMF1* mutation of patient 1 and his parents. Integrative genomics viewer presentation of the homozygous missense mutation in the *SUMF1* gene of patient 1 (exon4/9: c.529G > C, p.Ala177Pro), A. Sanger sequencing of *SUMF1* showing the base exchange G > C in parents a heterozygous state, B,C

Molecular testing revealed no pathogenic variants in *ARSA* or *PSAP*. Whole exome sequencing (WES) showed a homozygous missense variant in *SUMF1* (NM_182760.3) (c.529G > C, p.Ala177Pro) in patient 1 (not done in patient 2). Biallelic homozygous missense variants in *SUMF1* (c.529G > C, p.Ala177Pro) were confirmed in patient 1 and found in patient 2 by Sanger sequencing. Sanger sequencing revealed that both parents of each patient were heterozygous carriers (Figure 3).

### 3.4 Enzymatic and biochemical findings

Both patients showed severe deficiency of ARSA activity (0.00‐0.03 units/10^6^ leukocytes) in multiple tests (normal range 0.4 to 2.5 units/10^6^ leukocytes, mean 1.5 units/10^6^ leukocytes). Patient 1 additionally showed extremely low N‐acetyl‐glucosamine‐6‐sulfate‐sulfatase activity, but only partially reduced ARSB activity, and normal activities of N‐acetyl‐galactosamine‐6‐sulfate‐sulfatase, iduronate‐sulfatase and heparan‐N‐sulfatase (EC 3.10.1.1). ARSB and steroid‐sulfatase in patient 2 were normal (other sulfatases not measured). Test numbers, patients' ages, activity levels and normal ranges of respective sulfatases see Figure S2. Both patients showed high urinary excretion of sulfatides in multiple measures (Figure S3), but normal excretion of glycosaminoglycans. In patient 2, urinary excretion of oligosaccharides was normal and serum‐protein electrophoresis revealed increased α_2_‐globulin (14.7%) (normal range 7.2%‐11.3%).

## 4 DISCUSSION

Both MSD patients presented with clinical and neurological findings compatible with MLD like gait disorder, spasticity, tremor, ataxia, swallowing problems, and psychomotor regression resulting in loss of ambulation and loss of speech (Supplementary Material S1). Cerebral MRI showed a pattern of demyelination compatible with MLD (Figure S1),^37^ and electroneurography showed mixed axonal and demyelinating polyneuropathy. However, both children additionally suffered from dermal ichthyosis and were of short stature, symptoms which are not typical for MLD but suggest MSD.^12^, ^13^, ^38^ As MSD is a different disease than MLD despite the above mentioned similarities, other symptoms resembling different mucopolysaccharidoses could have been expected (see below). Focused on the patients´ symptoms, not all clinical assessments required clarifying MSD were done.^7^ First biochemical findings were compatible with MLD or MSD revealing severe ARSA deficiency in leukocytes and sulfatide accumulation in urine (Figures 2 and 3). As described in MSD, examination of other sulfatase activities revealed an inconsistent profile (Figure S2).^8^, ^9^, ^39^, ^40^ ARSB activity was only partially reduced in patient 1 and even normal in patient 2. In addition, normal activities of other sulfatases in both patients might explain the absence of hepatosplenomegaly, bone alterations, or marked coarse aspects in our patients. MLD and SAP‐B deficiency were genetically excluded. Finally, WES revealed a homozygous c.529G > C, p.Ala177Pro variant in *SUMF1* in patient 1. This was confirmed by Sanger sequencing in both patients (Figure 3). Critically reflected, wider assessment of sulfatases or earlier *SUMF1* sequencing would have led to a less delayed diagnosis of MSD.^7^ Early diagnosis prevents MSD patients from unhealthy therapy.

The *SUMF1* variant c.529G > C was absent in >4500 exome datasets of an in‐house database and public databases (gnomAD [10/2020]). It has been reported in only one MSD patient of Iranian/Dutch origin in compound heterozygosity with a frameshift‐variant (c.748delC) showing late‐infantile onset and a slightly more progressive course.^3^, ^39^ Homozygosity of the c.529G > C variant has not yet been reported. Functional studies in the meantime have confirmed the pathogenic potential of this variant.^41^ In their analysis of 20 patients with *SUMF1* mutations Cosma et al^3^ did not find a consistent genotype‐phenotype correlation, nor did this Hijazi et al^13^ in eight Saudi patients. A homozygous *SUMF1* stop‐mutation described in a baby with severe neonatal MSD suffering from hydrops fetalis^5^ could indicate that biallelic nonsense‐mutations are associated with a severe disease course. Adang et al^7^ report on poor outcome and decreased survival in MSD patients bearing two “severe”‐labeled variants (severe‐missense/nonsense‐alleles). In a recent meta‐analysis (75 publications including 143 MSD patients and 53 *SUMF1* mutations) Schlotawa et al^1^ demonstrated a correlation of survival and mutation severity but not sulfatase activities. Correlation of phenotype and enzyme deficiency seems lacking so far.^1^, ^2^, ^7^

To investigate whether dynamics in disease course may serve as an early clue for MSD in children presenting with symptoms of ARSA deficiency, we compared the clinical course of our two MSD patients (Supplementary Material S1) to a cohort of 59 (21 late‐infantile, 28 juvenile) patients with MLD regarding gross motor‐, speech‐, and swallowing decline by the use of standardized tools.^16^, ^17^, ^18^, ^29^ This approach was shown as essential describing natural history of neurodegenerative diseases.^7^, ^16^, ^18^, ^19^, ^20^, ^21^

Patient 1 and 2 showed first motor symptoms (GMFC‐MLD level 1) definitively later than 90% of patients with *late‐infantile* MLD do, but earlier than the earliest 10% of *juvenile* MLD.^18^ Thus, motor onset in both MSD patients occurred between what is described for late‐infantile and juvenile MLD. But progression then was rather slow as patient 1 lost independent walking (GMFC‐MLD level 2) at the age of 11 years, and patient 2 at the age of 9 years, corresponding the 75th percentile of juvenile MLD. Also, entry into GMFC‐MLD level 5 (loss of gross motor function, only head control preserved) was relatively slow, again corresponding to the second half of juvenile MLD. Time difference from GMFC‐MLD level 1 to level 2 was clearly longer than the 75th percentile of juvenile MLD (8 and 6 years vs 4.3 years). Time difference from GMFC‐MLD level 2 to level 5 corresponded to the 75th percentile of juvenile MLD in patient 2 and was even not reached in patient 1. This illustrates that the disease dynamics of the two MSD patients, despite of their earlier onset, was differently protracted in comparison to the well‐known disease dynamics in juvenile MLD (Figure 2).^18^, ^19^

First language decline in both MSD patients was relatively late compared with juvenile MLD (clearly after 50th percentile).^16^ But subsequent decline then was rather rapid and complete loss of expressive language (aged 14 years in patient 1 and 7.5 years in patient 2) occurred corresponding to the 25th and 50th percentile of juvenile MLD, respectively.^16^ Thus, concerning language decline, there was an almost inverse pattern of disease dynamic compared with motor function—starting later, but progressing more rapidly. This might support the idea that language decline is not only due to motor discoordination but also might indicate disabled speech‐concept in line with mental decline. Relevant swallowing problems (EDACS 2) in patient 2 occurred about 3 years after onset, corresponding to early cases of juvenile MLD (median 13 years after onset, range 2‐18 years)^19^, but did not yet occur in patient 1.

In a recent natural history analysis of MSD, Adang et al^7^ found characteristic constellations of “early developmental delay, ichthyosis, hepatosplenomegaly, and hearing loss.” Biallelic *SUMF1* mutations labeled as “mild” were associated with onset >1 months of age, achievement of independent ambulation and multiword sentences, slower regression, and longer survival.^7^ Our two MSD patients clearly belong to this milder group, however not prototypical as not suffering from organomegaly or deafness. This might be discussed in line with not yet described homozygosity of their mutation.

In summary, age of onset, occurrence of specific clinical signs, and genotype in MSD are recently discussed as key variables determining the outcome of the disease.^1^ In comparison to a broad cohort of patients with severe ARSA deficiency due to MLD, we demonstrated different dynamics of disease course in two patients with MSD. Although the here presented two individuals seem not entirely to represent the recently published characteristic pattern of MSD,^7^ we though propose disease dynamics as an additional clue for the characterization of MSD. Detailed description of long‐term course even of single individuals may contribute to natural course data of MSD. As an outlook, prospective natural history follow‐up is of urgent interest regarding counseling and further therapeutic options.

## AUTHOR CONTRIBUTIONS

Stefanie Beck‐Wödl performed NGS data analysis and did the molecular investigation. She was involved in drafting the paper. Christiane Kehrer wrote the paper. She did clinical investigation of the patients and communicated with their families. Klaus Harzer did substantial work in drafting the paper and supervised the laboratory work and communicative processes. Tobias B. Haack supervised the molecular work; Friederike Bürger and Dorothea Haas were responsible for the multiple sulfatase assays, GAG determinations, and GAG electrophoresis; Angelika Rieß did the genetic counseling of the families; Samuel Groeschel contributed and interpreted neuroimaging data and was involved in clinical investigation; Ingeborg Krägeloh‐Mann revised the manuscript critically and gave final approval of the version to be published; Judith Böhringer provided essential biochemical data of the patients, was responsible for the exchange of biochemical information. She did substantial work in drafting the paper. All authors critically revised the manuscript.

## COMPETING INTEREST

Stefanie Beck‐Wödl, Christiane Kehrer, Klaus Harzer, Tobias B. Haack, Friederike Bürger, Dorothea Haas, Angelika Rieß, Samuel Gröschel, Ingeborg Krägeloh‐Mann, und Judith Böhringer declare that they have no financial or personal relationships that inappropriately influence (bias) their actions (such as dual commitments, competing interests or competing loyalties) or might affect their scientific judgment. There are no financial relationships (such as employment, consultancies, stock ownership, honoraria, or paid expert testimony) nor personal relationships, academic competition, and intellectual passion which conflict to publication of this manuscript. SG received institutional research support from Shire, a Takeda company, outside of the submitted work. He is advisor and co‐investigator for trials in Metachromatic Leukodystrophy (Shire, Orchard), but receives no personal payment related to this role. Authors declare that there are no close relationship with, or a strong antipathy to, a person whose interests may be affected by publication of the article, academic link or rivalry with someone whose interests may be affected by publication of the article, membership in a political party or special interest group whose interests may be affected by publication of the article, deep personal or religious conviction that may have affected what the author wrote and that readers should be aware of when reading the article. Authors declare that they have not accepted the following from an organization that may in any way gain or lose financially from the results of their study or the conclusions of their review, editorial, or letter in the past 5 years: reimbursement for attending a symposium, a fee for speaking or for organizing education, funds for research or for a member of staff, a fee for consulting. Authors declare that they have not been employed by an organization that may in any way gain or lose financially from the results of their study or the conclusions of their review, editorial or letter in the past 5 years and that they have not held any stocks or shares in such an organization or have any other competing financial interests. Authors declare that they have not acted as an expert witness about their study, review, editorial, or letter. Authors declare that the submitted study has not been previously published and is not being considered for publication elsewhere nor submitted simultaneously to another journal. Authors declare that the work submitted is their own and copyright has not been breached in seeking its publication.

## ETHICS STATEMENT

The study was approved by the Ethical Committee of the University of Tübingen (Nr. 401/2005). This article does not contain any studies with animal subjects performed by the any of the authors. Approval from the Institutional Committee for Care and Use of Laboratory Animals (or comparable committee): not applicable.

## INFORMED CONSENT

All procedures followed were in accordance with the ethical standards of the responsible committee on human experimentation (institutional and national) and with the Helsinki Declaration of 1975, as revised in 2000 (5). Informed consent was obtained from all patients for being included in the study. Additional informed consent was obtained from all patients for which identifying information is included in this article.

## PATIENT CONSENT STATEMENT

Informed consent within the scope of the nationwide German research network LEUKONET was given by the parents of the two MSD patients including publication of patient‐related materials and MLD patients in all cases. Additional written informed consent was given for identifying video‐typing, and photographs.

## Supporting information


**Supplementary Figure 1** **Neuroimaging findings in patient 1 and 2 with multiple sulfatase deficiency**
T2‐weighted image of patient 1 at 9 years of age (6 years after first symptoms) showed diffuse white matter (WM) involvement (including lobar WM and corpus callosum) and atrophy, resulting in an MR severity score for MLD of 21. Corresponding T2‐weighted images of patient 2 at 5.5 and 16 years of age resulting in an MR severity score for MLD of 14 and 29 respectively demonstrate the progression of MRI changes and are compatible with juvenile MLD.^37^ T2‐weighted MRI images refer to coronal and sagittal images.

**Supplementary Figure 2** **Profile of sulfatase activities in blood samples from the two patients suffering from multiple sulfatase deficiency due to homozygous missense variant in *SUMF1* (c.529G > C)**
Values as % mean of normal enzyme activities. Gray bars indicate the normal range of the respective enzyme measurements. Arylsulfatase A, N‐Acetyl‐glucosamine‐6‐sulfatesulfatase, Arylsulfatase B, N‐Acetyl‐galactosamine‐6‐sulfate‐sulfatase, Heparan‐N‐sulfatase and Steroid‐sulfatase activities estimated in white blood cells, standardized by cell protein content or cell count; Iduronate‐sulfatase activity estimated in blood plasma, standardized by volume. In patient 1, enzyme activities of blood samples at the ages of 12.8 years (red crosses) and 14.3 years (red filled circles) are shown in the Figure S2 (additional single ARSA measurements revealing a similar enzyme activity at the ages 7.9 years, 9.3 years, and 10,8 years are not shown). In patient 2, ARSA measurements were performed in external hospitals at the ages of 5 (black asterisk) and 6 years (blue asterisk), ARSB and steroid‐sulfatase at the age of 6 years (blue asterisk).

**Supplementary Figure 3** **Urinary sulfatide (sulfoglycosphingolipid) excretion in patient 2 with multiple sulfatase deficiency**
Sulfoglycosphingolipids determined by two‐dimensional thin layer chromatography of urinary lipid extract. (Normal control urines usually show no distinct SU spots with this method in 24 hours collecting urine). Symbols: st = chromatographic start point of sulfatide standard; ST = chromatographic start point of patient urinary lipid extract; su = spots of sulfatide standard (two lipid‐chemical subtypes); SU = spots of patient's sulfatides from 10 mL urine; DHC = dihexosylceramide (major urinary glycosphingolipid also in normal controls); GSL = (other) glycosphingolipids; PL = phospholipids; GLC = glucose.

**Supplementary Table 1** see extra file

**Appendix** S1: Supporting Information
