# Supplementary figures and images for: Long‐term disease course of two patients with multiple sulfatase deficiency differs from metachromatic leukodystrophy in a broad cohort
Source: JIMD Rep. 2020 Dec 8;58(1):80–8. doi: 10.1002/jmd2.12189 (PMC7932862; doi:10.1002/jmd2.12189)

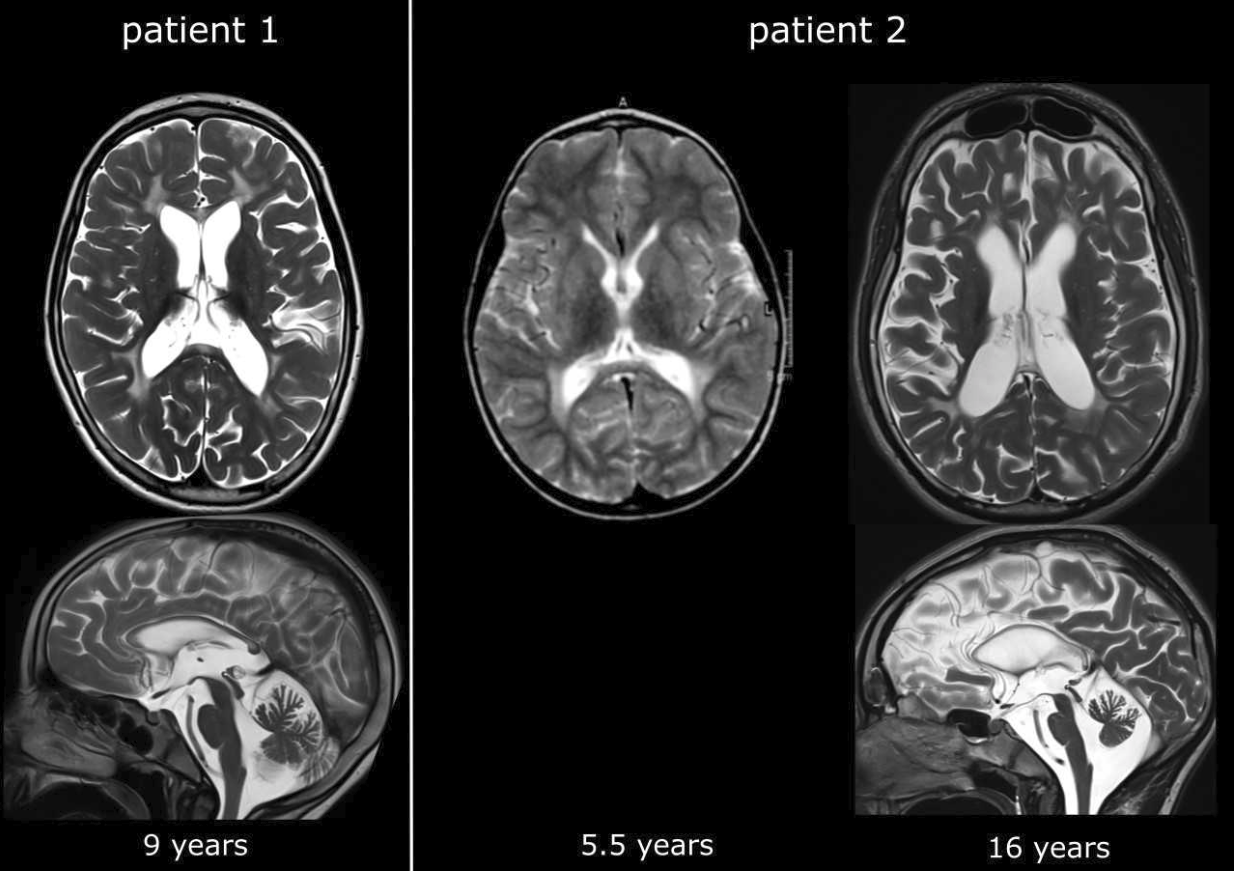

Supplement: Supplementary file 1 — Supplementary Figure 1 Neuroimaging findings in patient 1 and 2 with multiple sulfatase deficiency T2‐weighted image of patient 1 at 9 years of age (6 years after first symptoms) showed diffuse white matter (WM) involvement (including lobar WM and corpus callosum) and atrophy, resulting in an MR severity score for MLD of 21. Corresponding T2‐weighted images of patient 2 at 5.5 and 16 years of age resulting in an MR severity score for MLD of 14 and 29 respectively demonstrate the progression of MRI changes and are compatible with juvenile MLD.37 T2‐weighted MRI images refer to coronal and sagittal images. [file JMD2-58-80-s001.png]

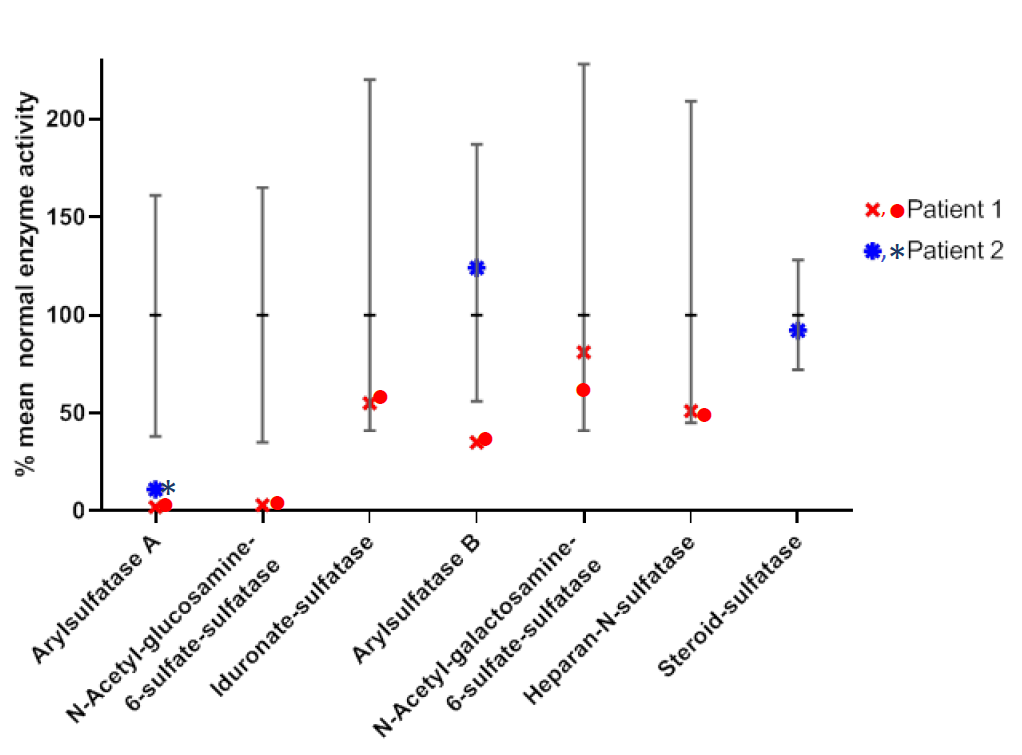

Supplement: Supplementary file 2 — Supplementary Figure 2 Profile of sulfatase activities in blood samples from the two patients suffering from multiple sulfatase deficiency due to homozygous missense variant in SUMF1 (c.529G > C) Values as % mean of normal enzyme activities. Gray bars indicate the normal range of the respective enzyme measurements. Arylsulfatase A, N‐Acetyl‐glucosamine‐6‐sulfatesulfatase, Arylsulfatase B, N‐Acetyl‐galactosamine‐6‐sulfate‐sulfatase, Heparan‐N‐sulfatase and Steroid‐sulfatase activities estimated in white blood cells, standardized by cell protein content or cell count; Iduronate‐sulfatase activity estimated in blood plasma, standardized by volume. In patient 1, enzyme activities of blood samples at the ages of 12.8 years (red crosses) and 14.3 years (red filled circles) are shown in the Figure S2 (additional single ARSA measurements revealing a similar enzyme activity at the ages 7.9 years, 9.3 years, and 10,8 years are not shown). In patient 2, ARSA measurements were performed in external hospitals at the ages of 5 (black asterisk) and 6 years (blue asterisk), ARSB and steroid‐sulfatase at the age of 6 years (blue asterisk). [file JMD2-58-80-s002.png]

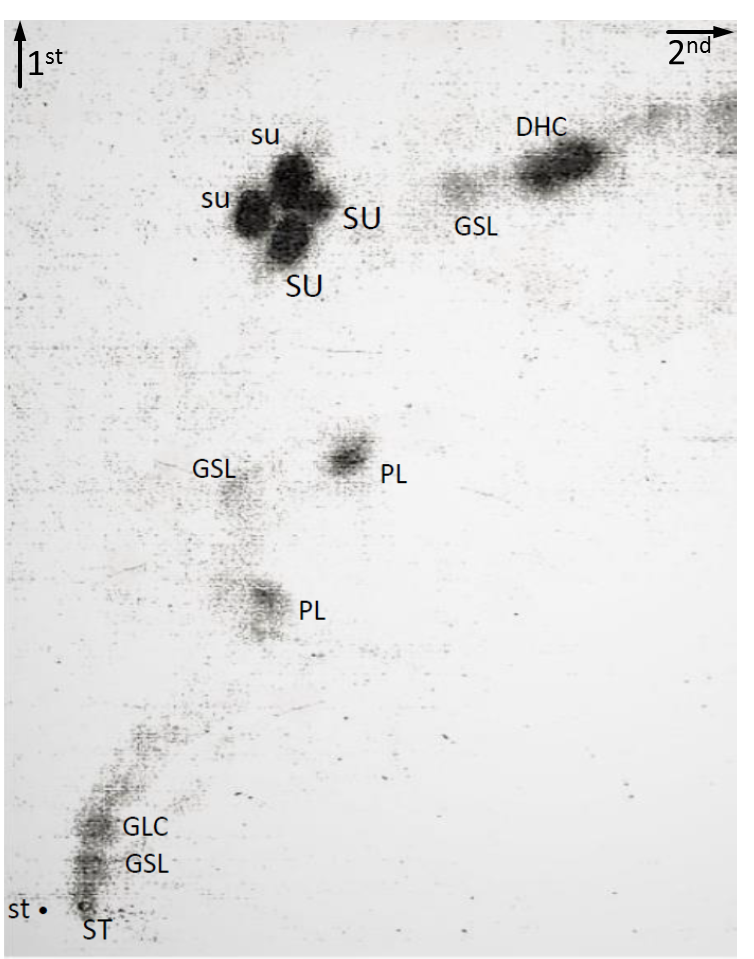

Supplement: Supplementary file 3 — Supplementary Figure 3 Urinary sulfatide (sulfoglycosphingolipid) excretion in patient 2 with multiple sulfatase deficiency Sulfoglycosphingolipids determined by two‐dimensional thin layer chromatography of urinary lipid extract. (Normal control urines usually show no distinct SU spots with this method in 24 hours collecting urine). Symbols: st = chromatographic start point of sulfatide standard; ST = chromatographic start point of patient urinary lipid extract; su = spots of sulfatide standard (two lipid‐chemical subtypes); SU = spots of patient's sulfatides from 10 mL urine; DHC = dihexosylceramide (major urinary glycosphingolipid also in normal controls); GSL = (other) glycosphingolipids; PL = phospholipids; GLC = glucose. [file JMD2-58-80-s003.tif]
