# Supplementary material for: Long‐term disease course of two patients with multiple sulfatase deficiency differs from metachromatic leukodystrophy in a broad cohort
Source: JIMD Rep. 2020 Dec 8;58(1):80–8. doi: 10.1002/jmd2.12189 (PMC7932862; doi:10.1002/jmd2.12189)
Supplement: Supplementary file 4 — Supplementary Table 1 see extra file [file JMD2-58-80-s004.docx]

Supplementary Table 1:

Electroneurographic findings of the two MSD patients

|  | Analyzed  nerves | Distal motor latency [ms] | Motor amplitude [mV] | Motor velocity [m/s] | Sensoric evoked potential, amplitude [mV] | Sensoric velocity [m/s] |
| --- | --- | --- | --- | --- | --- | --- |
| Patient 1**^a^**  (age 9.3 years) | N. medianus  N. tibialis  N. suralis | 8.9 (norm 3.2)  12.0 (norm 3.8)  - | 8.2 (norm 7.6)  0.1 (norm 4.0)  - | 13 (norm 54)  10 (norm 53)  - | 4.1 (norm 17.3)  -  - | 12 (norm 51)  -  - |
| Patient 1**^a^**  (age 14.3 years) | N. medianus  N. tibialis  N. suralis | 9.6 (norm 4.6)  -  - | 6.5 (norm 5.0)  -  - | -  -  - | -  -  - | -  -  - |
| Patient 2**^b^**  (age 6 years) | N. medianus | Distinctly increased | Reduced | Distinctly reduced |  | Distinctly  reduced |

**^a^** Electroneurography of the N. suralis was not feasible due to limited cooperation.

**^b^** No quantitative data are available (electroneurography performed in external hospital)
