# Supplementary material for: Long‐term disease course of two patients with multiple sulfatase deficiency differs from metachromatic leukodystrophy in a broad cohort
Source: JIMD Rep. 2020 Dec 8;58(1):80–8. doi: 10.1002/jmd2.12189 (PMC7932862; doi:10.1002/jmd2.12189)
Supplement: Supplementary file 5 — Appendix S1: Supporting Information [file JMD2-58-80-s005.docx]

**Supplementary Material 1: Detailed description of the patients suffering from multiple sulfatase deficiency due to homozygous missense change in *SUMF1* (c.529G>C)**

**Patient 1** (see Figure 1 A) was born spontaneously after uneventful pregnancy in the 37 5/7 week of gestation. Anthropometric data were normal for gestational age (birth weight 2725 g (10^th^ percentile), birth length 48 cm (25^th^ percentile), head circumference 33 cm (10^th^ percentile). Her early infantile development was within the normal range. She was able to sit and to crawl at the age of 10 months (90^th^ percentile) and learnt to walk independently at the age of 17 months (90-97^th^ percentile).^42^ Language acquisition was normal for her native language (50^th^ percentile) but showed some delay in the German language due to bilingual education).^42^ At the age of 3 years, she was able to speak in short sentences in both languages. Between the age of 30 and 36 months, a supination of her feet was noted, followed by gait disturbances, and walking on tiptoes (GMFC-MLD 1). A routine paediatric investigation at the age of 5 years revealed tiptoeing gait, elbows in flexed position, combined with mild fine motor problems due to tremor, but also slight first cognitive delay. At the age of 5 ½ years, progress in language ceased, and deterioration in speech occurred within the sixth year of life (ELFC-MLD 1). She went to a school for physically and mentally disabled children. Neurological signs such as increased muscle tone in arms and legs, hypotonia of the trunk, diminished and then absent muscle reflexes, but also tremor, ataxia, and problems in swallowing became more and more obvious within the following years. With respect to her development, cognitive and speech-related problems were the predominant signs. At the age of 7 to 8 years, the ability to speak in whole short sentences was lost, and the girl used only two or even single words (ELFC-MLD 2). At the age of 14 years, loss of language occurred (ELFC-MLD 4). Limitation in understanding language was observed at the age of nearly 11 years. Fine motor function was hampered by increasing tremor, and at the age of 9 years, complex bimanual function was restricted (Bimanual Fine Motor Function Measure (BMFM) 2). At the age of 11 years, independent walking was lost (GMFC-MLD 2) and walking with hold on two hands was lost at the age of 14 years (GMFC-MLD 3). Currently, at the age of 15-6/12 years, standing is hampered due to progressive clump foots and the ability to sit freely is lost, but crawling or locomotion on the floor is still possible (GMFC-MLD 4a). GMFM-88 testing revealed a total score of 75.5 % at the age of 9 years, a total score of 61.1 % at the age of 10-10/12 years, a total score of 43% at the age of 12-10/12 years, and a total score of 34,8 % at the age of 15-6/12 years reflecting motor decline. At the age of 15-6/12 years, some limitations to efficiency in eating and drinking (EDACS 1) occurred. At this time, short stature (body length 128 cm (22 cm <3^rd^ percentile)) and underweight (weight 22 kg (18 kg <3^rd^ percentile)) were documented, but head circumference was normal (52 cm (10^th^ percentile)). There were no relevant or obvious constraint by visual or hearing problems, cardiorespiratory problems, or scoliosis. As an additional somatic sign there was dermatologically confirmed diagnosis of ichthyosis since the age of about five years but ameliorating over time.

**Patient 2** was born at term after uneventful pregnancy. Anthropometric data were within the lower range (birth weight 2945 g (6^th^ percentile), birth length 49 cm (6^th^ percentile), and head circumference 34 cm (11^th^ percentile). His early motor development was on the borderline range; he was able to sit and to crawl at the age of 10 to 11 months (90^th^ percentile) and he learnt to walk independently at the age of 18 months (97^th^ percentile) ).^42^ Language acquisition was normal (50^th^ percentile) (first words with 12 months, two-word sentences with 24 months, whole sentences with 30 months)).^42^ Falling and stumbling as well as unsteady gait was noted at the age of about 3 years (GMFC-MLD 1). Due to mild ataxia, walking on stairs was no longer possible. He sought stabilization by holding his arms flexed. At the age of 5 years, intention tremor and dysarthria occurred. Weakness in trunk and lower extremities was a predominantly neurological sign, but also stiffness of the limbs due to central nerve-damage and touch-sensitivity due to peripheral neuropathy became obvious. Dysmetria, absent muscle reflexes, and swallowing problems followed (EDACS 2 and 3). Dementia developed between the age of 5 to 8 years, and he went to school for disabled children. Language declined at the age of 6.5 years (ELFC-MLD 1) and entry into ELFC-MLD 2 (two-words-sentences) occurred at the age of 7 years, and into ELFC-MLD 3 (single words) just after 7 years. Complete loss of language occurred at the age of 7.5 years. At the age of 10 years, only grasping and holding were possible (BMFM 3). He lost free walking at the age of 9 years (GMFC-MLD 2); and the ability to walk with support at the age of 10 years (GMFC-MLD 3), followed by the loss of the ability to sit or crawl within only a few months (GMFC-MLD 5). Head control was diminished, but not completely lost. He still had some minimal communication abilities with caregivers. As additional somatic signs, there was dermatologically confirmed diagnosis of ichthyosis since the age of 11 months but ameliorating over time. Epilepsy occurred at the age of 13 years, as well as a severe neuropathic scoliosis (see Figure 1 B). Relevant visual or hearing problems were not reported. At the age of 15 years, he was unable to eat and drink safely (EDACS 4) and got PEG-tube-fed. At this time, he was undersized (3^rd^ percentile) and showed severe underweight (27 kg <3^rd^ percentile). He died from pneumonia at the age of 20 years.

**Reference:**

**42**. Largo RH, Molinari L, Weber M, Comenale Pinto L, Duc G. Early development of locomotion: significance of prematurity, cerebral palsy and sex. Dev Med Child Neurol. 1985;27(2):183‐191.

**Supplementary Material 2: Description of the patients suffering from multiple sulfatase deficiency due to homozygous missense change in *SUMF1* (c.529G>C) referring to data of the natural history of MSD provided by Adang et al.^7^ with regard to a) classification terms, b) neurological exam features, c) somatic exam features, and d) medical complications of MSD.**

1. **Classification terms:**

Classification of MLD into a “late-infantile” form with age of onset <30 months and a “juvenile” form with age of onset between 2,5 and 15 years (<16 years) refers to former natural course studies in MLD^16,18,19^ and must not mixed up to a classification into a “neonatal”, “severe”/”mild”-infantile, or “juvenile” form of MSD.^4,15^ But currently a classification with regard of the disease onset in MSD is proposed as <1 months versus >1 months of age, the latter covering the here presented patients 1 and 2.^7^

**b) Neurological exam features:**

- Motor abnormalities were observed in patient 1 since the age of 30 and 36 months, and in patient 2 since the age of about 36 months.

- Tone abnormalities were observed in patient 1 since the age of 5 years, and in patient 2 since the age of 3-5 years.

- Ataxia or tremor were observed in patient 1 since the age of 5 years, and in patient 2 since the age of 3 years (ataxia) (5 years tremor).

- Language abnormalities were observed in patient 1 since the age of 6 years, and in patient 2 since the age of 6,5 years.

- Behavioural abnormalities were observed in patient 1 since the age of about 6 years, and in patient 2 since the age of 5 years.

- Microcephaly was not observed (neither patient1 nor patient 2).

- Peripheral neuropathy was seen in patient 1 and 2 since the age of about 7 years (clinically/ first EMG of external hospital).

**c) Somatic exam features:**

- “Dysmorphic features”, “abnormal hearing evaluation”, “abnormal eye exam”, “abnormal cardiac evaluation”, “abnormal respiratory exam” and “hepatosplenomegaly” were not found (partly not assessed, but not clinically observed) in both patients.
- Ichthyosis was found in both patients, in patient 1 since the age 5 years and in patient 2 since the age of 11 months.
- “Decreased range of motion of joints” was observed in both since the age of around 6 to 8 years (timepoint can´t be exactly given).
- Scoliosis was only observed in patient 2 since the age of about 13 years.

1. **Medical complications of MSD:**

- “Respiratory support”, “hearing aid placement”, and “otitis media” weren´t mentioned in both patients.
- “Feeding tube initiation” took place at the age of 15 years in patient 2 but is not yet necessary at the age of 15,5 years in patient 1.
- Age of onset of “urinary incontinence” can`t be exactly given in both patients, probably, there was primary (nocturnal) incontinence.
- “Seizure onset” was at the age of 13 years in patient 2 and not yet seen in patient 1.
- “Orthopaedic complication” was observed since the age of about 5 years in both patients.

**Supplementary Material 3: Description of urinary glycosaminoglycan (GAG) analysis and urinary lipid analysis:**

1. Urinary glycosaminoglycan (GAG) analysis:

Whole fraction of urinary glycosaminoglycans was separated electrophoretically into the single types that were estimated for their relative percentages.

1. Urinary lipid analysis:

5-10 ml of 24-h-urine portion was filtered through Whatman no. 10311652 paper.^35^ The air-dried filter paper was extracted with 10 ml chloroform / methanol (2:1) for 2 h. The extract was phase-partitioned by admixing each 10 ml methanol and water and shaking. The cleared upper phase was discarded. The lower phase together with turbid supernatant was dried with warm airflow. The dry residue was dissolved in 0.5 ml chloroform / methanol (2:1), concentrated to a small volume, and applied close to the lower left corner of a 10 x 10 cm thin-layer, HPTLC plate (Merck, Darmstadt, Germany, no. MC10506130001). The sulfatide standard from MLD kidney was applied 5 mm to the left of the urine extract application point. Further chromatographic and lipid staining techniques were performed according to Hörtnagel et al.^36^

**Supplementary Table 1:** see extra file

**Supplementary Weblinks and URLs**

NGS pipeline https://github.com/imgag/megSAP

ExAC http://exac.broadinstitute.org/

gnomAD http://gnomad.broadinstitute.org/
